# Mechanical Behavior of Conventional and Subtractively Manufactured Dental Resin Composites After Water Degradation

**DOI:** 10.3390/bioengineering13070829

**Published:** 2026-07-17

**Authors:** Georgiana Osiceanu, Roxana Diana Vasiliu, Flavia Roxana Bejan, Radu Negru, Nicușor Alin Sîrbu, Raluca Faur, Liliana Porojan

**Affiliations:** 1 Department of Dental Prostheses Technology (Dental Technology), Center for Advanced Technologies in Dental Prosthodontics, Faculty of Dental Medicine, “Victor Babes” University of Medicine and Pharmacy Timisoara, Eftimie Murgu Sq. No. 2, 300041 Timisoara, Romania; roxana.vasiliu@umft.ro (R.D.V.); flavia.toma@umft.ro (F.R.B.); sliliana@umft.ro (L.P.); 2 Doctoral School, Faculty of Dental Medicine, “Victor Babes” University of Medicine and Pharmacy Timisoara, Eftimie Murgu Sq. No. 2, 300041 Timisoara, Romania; 3 Department of Mechanics and Strength of Materials, Politehnica University Timisoara, 300006 Timisoara, Romania; radu.negru@upt.ro; 4 National Research & Development Institute for Welding and Material Testing ISIM Timisoara, Blv. Mihai Viteazu 30, 300222 Timisoara, Romania; asirbu@isim.ro (N.A.S.); rfaur@isim.ro (R.F.); 5 Faculty of Chemical Engineering, Biotechnology and Environmental Protection, Politehnica University of Timisoara, Bld. Vasile Parvan 6, 300223 Timisoara, Romania

**Keywords:** dental materials, CAD-CAM, direct resin composite, flexural strength, 3-point bending test, elastic modulus, mechanical properties, fractography, SEM evaluation

## Abstract

The ongoing development of subtractive options for indirect restorations places clinicians in a position that requires adaptation, understanding and choosing of the most suitable option for the long-term survival of the restoration. Mechanical parameters represent important indicators of long-term success. Consequently, this research aimed to assess the impact of water sorption on the mechanical properties of two direct resin composites, Gradia Direct Anterior A2 and Filtek Z550 A2 and three CAD/CAM subtractively manufactured dental resin composites, Vita Enamic, Brilliant and Cerasmart. A total of one hundred specimens (50 control, 50 underwent this protocol: dehydration, immersion in distilled water for 30 days and then re-desiccation), standardized to the dimensions of 14 mm × 4 mm × 1.2 mm were subjected to three-point bending test (based on ISO 4049:2019 and ISO 6872:2015), in order to find out the flexural strength and the elastic modulus of the material at the breaking point. Then, the fractured sample surfaces were fractographical analyzed. Using the two-parameter Weibull approach, the Weibull modulus (m) and the characteristic strength (σ_0_) were evaluated. In this investigation, the elastic modulus varied from 5.8 (Gradia Control) to 20.31 (Vita Degraded) GPa, with the upper limit close to the values of natural dentin (17.7–29.8 GPa). The values of flexural strength ranged from 174.47 (Brilliant Control) to 79.2 (Gradia Control), subtractively processed materials demonstrating higher flexural strength and elastic modulus values than the direct resin composites, which is related to their high inorganic filler content. All material groups, except for Gradia Control, had a flexural strength more than the 80 MPa minimum value needed to sustain masticatory force. The dehydration and hydration cycles did not have a statistically significant influence on the mechanical properties of the material. The fractographic analysis revealed fracture patterns and features associated with the microstructure, with the PICN category material being particularly notable. Weibull analysis revealed that the direct resin composite materials exhibited higher reliability, lower data scatter and CAD-CAM materials showed greater characteristic strength, with a notably high performance of the nano-hybrid direct resin composite.

## 1. Introduction

In modern dentistry, a successful dental restoration should combine high mechanical strength with a natural appearance that meets patients’ aesthetic expectations [1].

Direct resin composites are conventional materials used for restoring cavities and consist of a resin matrix—a combination of monomers and photopolymerization initiators—that forms the organic phase and fillers of varying sizes, which constitute the inorganic phase [2].

In recent years, because of advancements in dentistry, CAD/CAM (Computer-Aided Design/Computer-Aided Manufacturing) has developed as a suitable option for indirect restorations, which include fixed prosthodontic (crowns, veneers, and dental bridges) [3]. Subtractive manufactured CAD/CAM materials can be classified into ceramics, metals, resins, such as polymethyl methacrylate (PMMA), resin composites, and nanoceramics, as well as polymer-infiltrated ceramic-network (PICN) materials (for example, Vita Enamic) [4].

Resin composite blocks consist of a proportion of inorganic fillers, such as silica and zirconia, combined with monomers, that undergo industrial polymerization and are thought to show good mechanical properties and reduced brittleness, due to their dual polymer-ceramic character [5].

The main distinct characteristics of ceramic and resin composites lie in their different chemical compositions and internal structures, which influence their mechanical properties. Ceramics are known to show significantly elevated flexural strength values [6]. However, CAD/CAM resin composites have the potential to mix the advantageous mechanical, aesthetic, and physical qualities of composites and ceramics [5].

Assessing the mechanical properties of the dental material (such as microhardness, elastic modulus, and flexural strength) is important because these parameters act as predictors of clinical behavior [7]. Factors that may influence mechanical strength include the dimensions and arrangement of the particles, as well as the distribution and quantity of the resin matrix monomers [8,9,10,11]. Nevertheless, other crucial components are the base and diluting monomers, which, in accordance with reduced polymerization shrinkage, influence the amount of water absorbed by the resin monomer structures [12]. Because water sorption causes inert monomers to diffuse into the oral environment, the filler component to separate and the mechanical qualities to deteriorate over time, it may therefore have a detrimental impact on the restoration’s long-term longevity [12].

The test that examines the mechanical parameters by combining compressive, tensile, and shear stresses is the three-point bending test [13]. These stress types are also considered in other mechanical testing methods, such as tensile testing of dental alloys coated with dental restorative materials [14,15]. Flexural strength testing on bar-shaped specimens is commonly adopted as a substitute for direct tensile testing [16]. The definitions of flexural strength and elastic modulus are: flexural strength describes the tension at which a material breaks when subjected to bending, while elastic modulus represents the rigidity of a material during bending [17].

From a biomimetic standpoint, the materials used for direct and indirect dental restorations need to demonstrate mechanical characteristics that closely resemble those of human dental hard tissues, enamel and dentin [18,19,20]. An optimal resin composite should combine an elastic modulus similar to that of dentin with the fracture toughness of ceramics [21].

Fractography is the art and science of analyzing fractured surfaces to identify specific features, such as the fracture origin, associated with mechanical testing for fatigue assessment, fracture resistance, dental wear, or flexural strength [22]. Its use in the field of dental materials dates back to 1989, but it gained significant attention around 2005 [23,24].

Qualitative fractography implies identifying and interpreting characteristic features on a fractured surface, such as the fracture origin, the direction of crack propagation, Wallner lines, fracture mirror and mist regions, hackle marks, the compression curl [25,26]. These features help the fractographer obtain valuable information about the cause of the fracture and the failure mechanism of a specific material.

Two types of fracture patterns are commonly recognized: brittle fracture, characterized by crack development, and ductile fracture, which involves quasiplastic deformation [27]. Resin composites can be classified as materials with plastic behavior due to the presence of their polymeric matrix.

Nevertheless, highly loaded resin composites are considered an intermediate category between ceramics and polymers. In addition, their fractographic characteristics are not easy to discern because of the rough microstructure present on the fracture surface [28]. Consequently, features such as hackle or Wallner lines may be hidden by the pronounced surface roughness induced by the reinforcing filler particles [16].

However, resin composites can also be considered brittle materials and can be analysed according to the mechanisms of brittle fracture, because of their high inorganic filler content, which enhances their mechanical properties and allows them to be used in areas subjected to high mechanical loads [29,30].

Thus, taking into account the potential effects of water immersion aging, this study aimed to compare the mechanical properties of direct resin composites with those of CAD/CAM materials, both unstored and stored in distilled water and also, to provide a fractographic perspective.

There were three null hypotheses implied in this study: (H1) There is no difference the flexural strength and the elastic modulus between the CAD-CAM resin composite groups and the direct resin composite groups post hydration-dehydration cycles; (H2) there is no difference between the two groups, from the same material (control, degraded); (H3) there are no fractographic differences between the examined samples.

## 2. Materials and Methods

### 2.1. Materials, Sample Preparation, Testing Protocol and Mechanical Parameters Calculation

In this study 5 materials from 2 categories were used: direct resin composite Gradia Direct (GC Corporation, Tokyo, Japan), Filtek Z550 (3M Espe, St. Paul, MN, USA) and subtractively processed CAD-CAM materials: Vita Enamic, Cerasmart, and Brilliant Crios. One hundred samples were prepared, with a dimension of 14 × 4 × 1.2 mm and were randomly divided into 2 groups: 50 control and 50 degraded. The surface of the samples was finished with abrasive papers with grit sizes ranging from 400 to 2000 and polished with Compo + polishing paste (Feguramed GmbH Buchen Germany).

Prior to testing, the 50 degraded samples followed a hydration-dehydration protocol: the specimens were placed in glass vials containing moisture-indicating silica gel and stored in an incubator at 37 °C (+/−1 °C) until a constant mass was achieved (mass loss of less than 0.1 mg over 24 h, achieved in 2–3 consecutive weeks). After that, they were immersed in 37 °C distilled water for a total of 30 days and then subjected to a second dehydration cycle until a final constant mass. The water was changed daily to avoid contamination.

**Figure 1:**
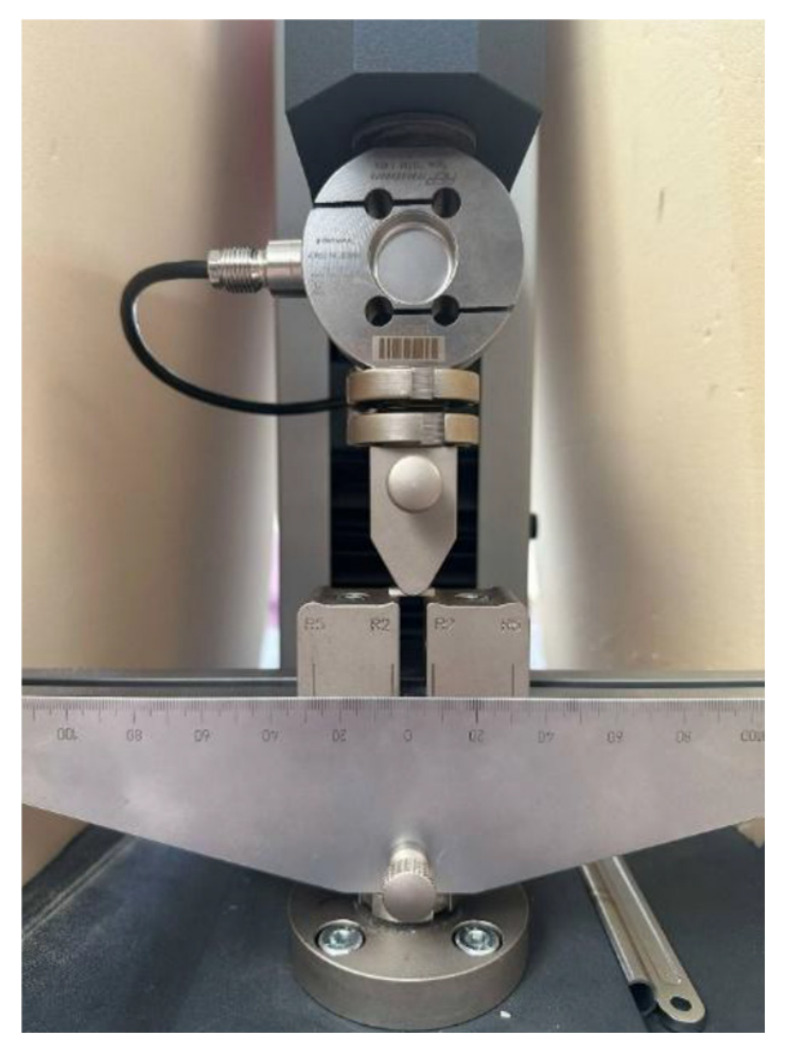
The three-point bending test machine with the sample positioned.

To evaluate mechanical resistance and the effect of hydration–dehydration cycles, the samples were subjected to a three-point bending test following ISO 4049 [31] and ISO 6872 [32] standards, using the universal testing machine for static loading Metrotec (Techlab Systems S.L., Lezo, Spain), equipped with a three-point bending setup and a 1000 N load cell. The support span length was set at 12 mm and the controlled speed was set at 1 mm/min (Figure 1). The maximum force before breaking was registered in order to calculate the flexural strength and elastic modulus.

The testing configuration described in ISO 6872 was chosen to ensure standardized testing conditions across all CAD/CAM materials, allowing direct comparison of their flexural properties. Although the specimen dimensions varied from those given in the original ISO standards, due to the size limitations of the CAD/CAM blocks, the testing technique conformed to the same mechanical principles and was performed through a mini-flexural configuration, as often applied in anterior research on both direct and CAD/CAM resin composites [32,33,34,35].

The composition and the manufacturer of the materials used in the study can be observed in Table 1 and Table 2.

The following formulae (1) and (2) were used to calculate the elastic modulus (E) and flexure strength (σ) at the highest flexure/fracture load on the load–displacement curve:

(1) E = FL^3^/4dwt^3^

(2) σ = 3FL/2wt^2^

F—the maximum flexure load (N), L—the length of the support span (12 mm), w—the width of the specimen (4 mm), t—the thickness of the specimen (1.2 mm), and d—the deflection at the load F (mm).

### 2.2. Fractography

Following the fracture assessment, the fracture surfaces were analyzed by scanning electron microscopy (SEM), using VEGA LMU scanning electron microscope (TESCAN, Brno, Czech Republic), at a magnification of 48× and 100× and observation scales of 1 mm and 100 and 200 µm.

### 2.3. Weibull Analysis

Weibull analysis was performed to determine two important parameters for failure risk evaluation: the characteristic fracture strength (σ_0_) and the Weibull modulus (m).

The specimens’ flexural strength values (Smax—MPa) were arranged in ascending order of strength, and the probability of failure (Pf) for each specimen was calculated using this formula [36]:

(3) P_f,i_ = n_i_ − 0.5/N

where n_i_ is the rank of the specimen in ascending order of the flexural strength (ex: n_i_ = 1, 2…N samples) and N is the total number of samples tested.

The cumulative probability of failure in accordance with Weibull statistics was calculated using the following equation:

(4) Pf = 1 − exp[−(σ/σ_0_)^m^]

where Pf = probability of failure, σ—characteristic flexural strength, m—Weibull modulus.

The Weibull modulus (m), calculated from the slope of the regression line, describes the material’s behaviour; higher values of the Weibull modulus suggest more constant behaviour, and lower values are associated with greater dispersion of the measured strength data [37]. For brittle materials such as ceramics the Weibull modulus (m) is considered one of the most important parameters and its value may be influenced by the filler content, particle dimensions and the mechanical strength testing method used [38,39].

The characteristic strength (σ_0_) represents the stress at which 63.2% of specimens are predicted to fail according to the Weibull distribution.

### 2.4. Statistical Analyses

Statistical analyses were performed using the JASP software (version 0.95.4). Data normality was assessed using the Shapiro–Wilk test, and homogeneity of variances was evaluated using Levene’s test. One-Way ANOVA was applied in order to assess if there are statistical significant differences (*p* < 0.05). Statistical differences between the control and deteriorated groups were analyzed using an unpaired Student’s *t*-test. Tukey Post-Hoc was applied, to check which material differs from another. Correlations between the elastic modulus and maximum force were evaluated using Pearson’s correlation coefficient, with these interpretations: 0–0.2 (“very weak”), 0.2–0.4 (“weak”), 0.4–0.6 (“moderate”), 0.6–0.8 (“strong”), and 0.8–1.0 (“very strong”).

## 3. Results

### 3.1. Flexural Strength—Means and SD

**Figure 2:**
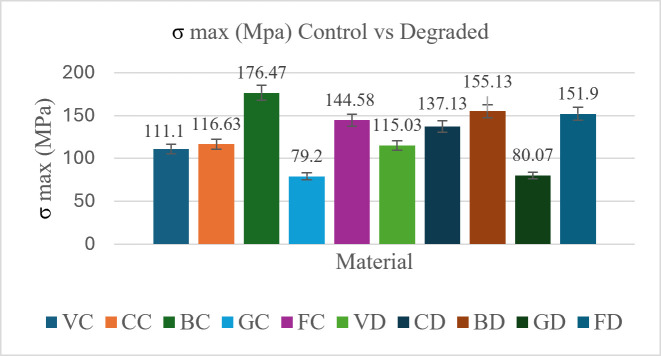
Means and SD of σ max for VC—Vita Control, CC—Cerasmart Control, BC—Brilliant Control, GC—Gradia Control, FC—Filtek Control, VD—Vita Degraded, CD—Cerasmart Degraded, BD—Brilliant Degraded, GD—Gradia Degraded, FD—Filtek Degraded.

In Figure 2, the means and standard deviations of σ max for the five materials can be seen. The highest value was recorded for Brilliant Control (174.47), while the lowest was recorded for Gradia Control (79.2).

### 3.2. Flexural Strength—Pairwise Comparisons

For pairwise comparisons between materials, the Tukey HSD post hoc test was applied. The statistically significant differences identified among most materials are presented in Table 3 (*p* < 0.05).

In case of the control groups, significant differences were found between almost all pairwise combinations, except for Vita-Cerasmart Control (*p*-0.959).

Regarding the degraded groups, Vita differs significantly from almost all other materials (Table 4). Differences among Cerasmart, Brilliant, Filtek, and Gradia are not statistically significant at 0.05. Significant differences were observed among nearly all pairwise comparisons, with the exception of Vita-Cerasmart Degraded, Cerasmart-Brilliant Degraded, Cerasmart-Filtek Degraded, and Brilliant-Filtek Degraded.

### 3.3. Elastic Modulus—Means and SD

**Figure 3:**
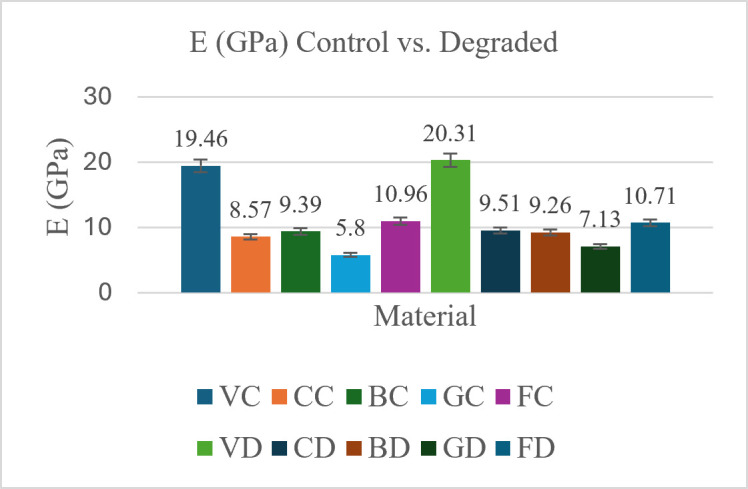
Means and SD of E for VC—Vita Control, CC—Cerasmart Control, BC—Brilliant Control, GC—Gradia Control, FC—Filtek Control, VD—Vita Degraded, CD—Cerasmart Degraded, BD—Brilliant Degraded, GD—Gradia Degraded, FD—Filtek Degraded.

Regarding the elastic modulus, the means and standard deviations are observed in Figure 3. The highest value was registered by Vita Degraded (20.31), while the lowest was recorded for Gradia Control (5.8).

### 3.4. Elastic Modulus—Pairwise Comparisons

Regarding the comparison of the elastic modulus values for the control groups, the values can be observed in Table 5. Vita and Gradia are each significantly different from all other groups, while Cerasmart, Brilliant, and Filtek do not differ significantly from each other. Significant differences were found between almost all pairwise combinations, except for Brilliant-Cerasmart Control and Brilliant-Filtek Control.

Regarding the comparison for the degraded groups, the values can be observed in Table 6.

Brilliant, Cerasmart, and Filtek are statistically similar to each other—no significant differences among these three.

### 3.5. Student’s t-Test and Pearson Correlation

The unpaired Student’s *t*-test (Table 7) revealed no significant differences between the two states (control vs. degraded), except for Gradia, which had a *p*-value of 0.001.

**Figure 4:**
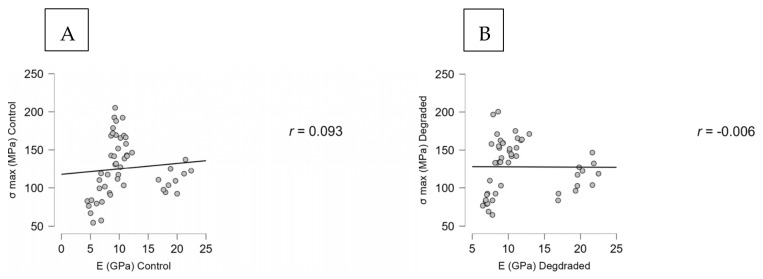
Pearson’s correlation between σ max and E control (**A**); σ max and E degraded (**B**).

The Pearson’s correlation revealed a very weak positive correlation (r: 0.093) between the σ max and E Control values and a very weak negative correlation (r: −0.006) between the σ max and E Degraded values (Figure 4).

### 3.6. Fractography Results

**Figure 5:**
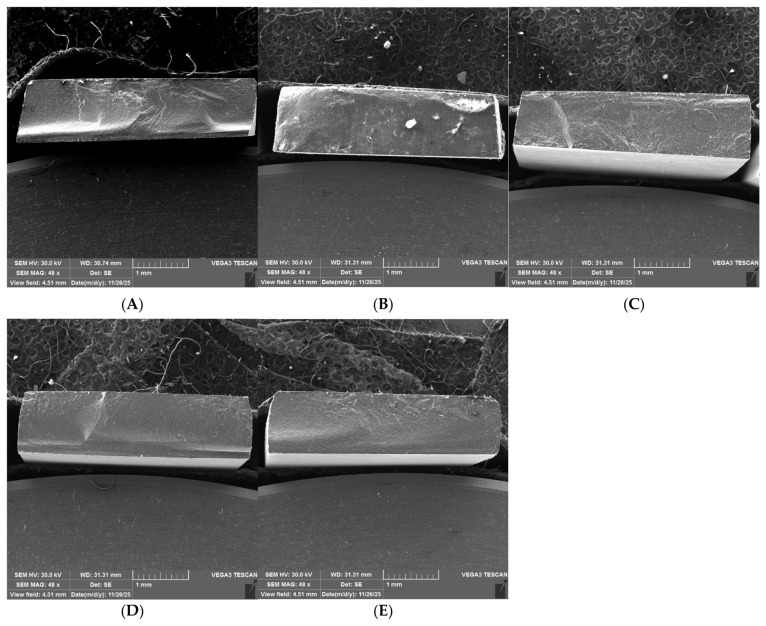
SEM images of representative fracture surfaces at 48× magnification: Filtek Z550 (**A**), Gradia Degraded (**B**), Gradia Control (**C**), Vita Degraded (**D**), Vita Control (**E**).

At 48× magnification, the representative SEM images revealed cracks, voids, and structural defects distributed within the investigated materials (Figure 5). Filtek Z550 (Figure 5A) presented an irregular fracture morphology with visible surface defects and heterogeneous topography. Gradia Degraded (Figure 5B) showed a rough fracture surface with dispersed irregularities, while Gradia Control (Figure 5C) exhibited a comparatively simpler fracture morphology with reduced surface fragmentation. In contrast, Vita Degraded (Figure 5D) demonstrated a more homogeneous and compact fracture surface with fewer visible defects, suggesting a more uniform internal microstructure. No major differences were observed between the control and degraded groups.

**Figure 6:**
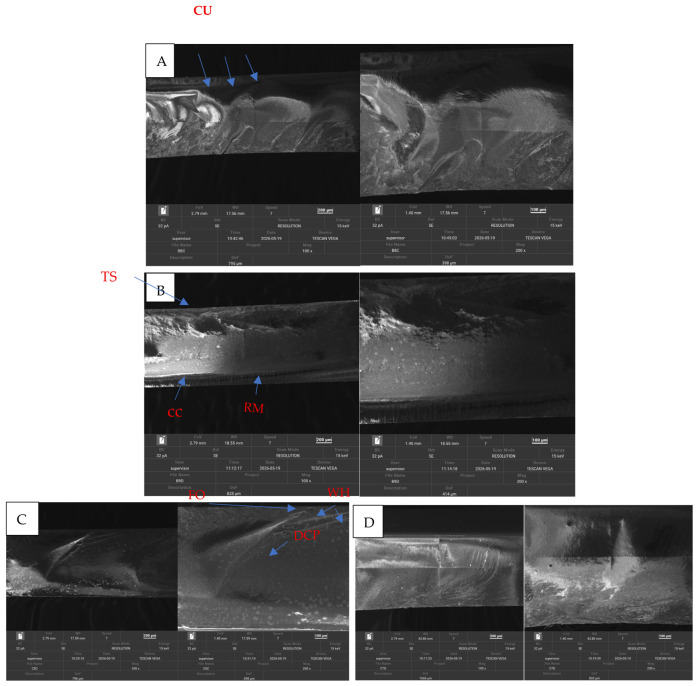

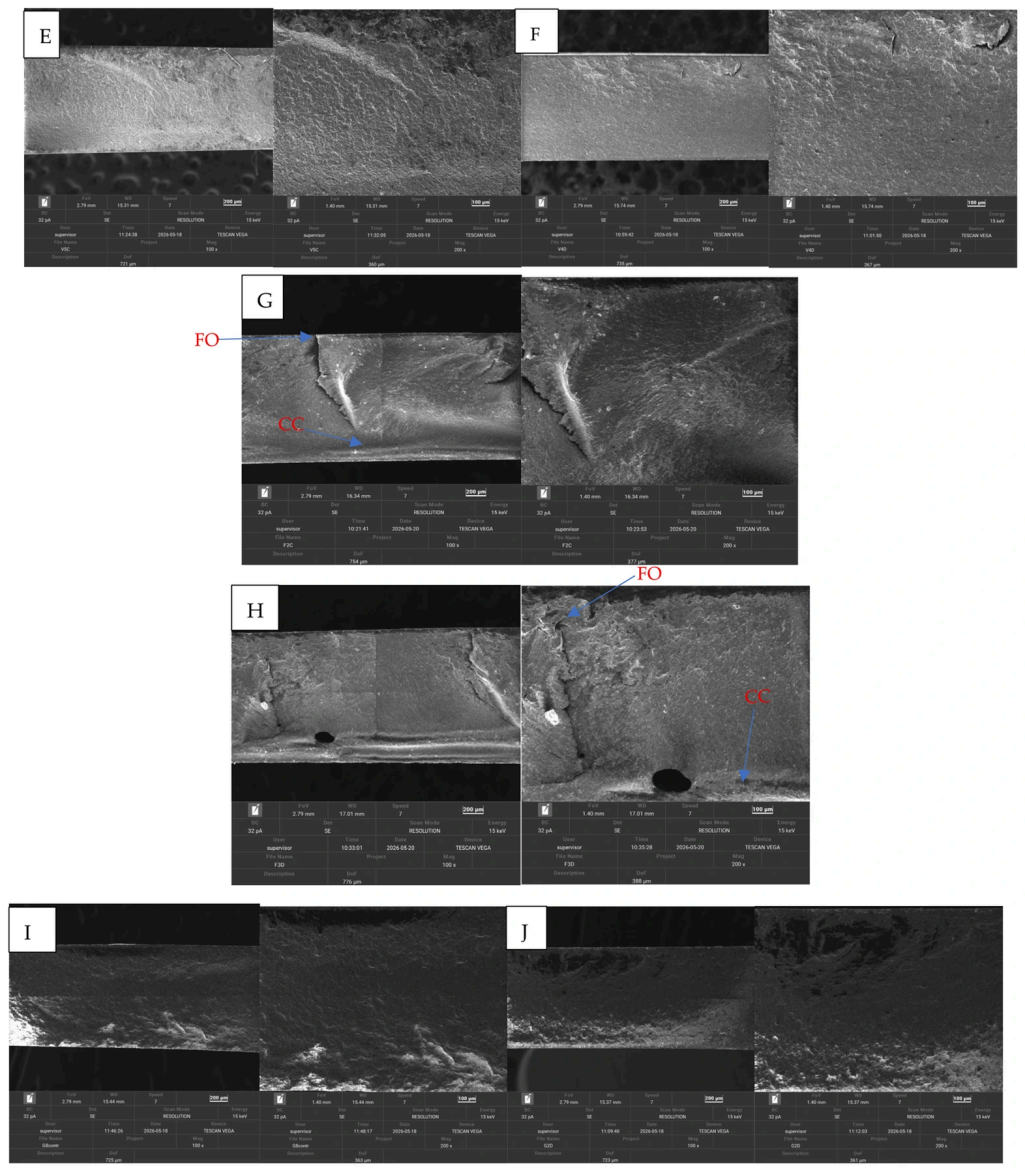
Brilliant Control (**A**), Brilliant Degraded (**B**), Cerasmart Control (**C**), Cersmart Degraded (**D**), Vita Control (**E**), Vita Degraded (**F**), Filtek Control (**G**), Filtek Degraded (**H**), Gradia Control (**I**), Gradia Degraded (**J**); CU—crack unions, RM—river markings, FO—fracture origin, CC—compression curl, DCP—direction crack propagation, WH—wake hackle, TS—tensile side.

Evaluation at 100× and 200× magnification of representative samples from each material revealed additional fracture-related surface details associated with crack initiation and propagation during the three-point bending test (Figure 6). In the Brilliant Crios Control sample (Figure 6A), curved and step-like ridge features are present on the fracture surface and may correspond to crack unions (coalescence ridges), generally aligned with the probable direction of crack propagation, according to the literature [40]. Features resembling river markings were identified in the Brilliant Crios Degraded sample (Figure 6B). Probable fracture origins could be identified in some samples, generally located on the tensile side of the specimen, while the apparent crack propagation direction extended away from these regions. Compression curl regions were also observed on the side opposite to the tensile zone (Figure 6C,D). Cerasmart samples (Figure 6E,F) showed heterogeneous fracture surfaces with localized irregularities and ridge-like formations, which suggest crack deflection around filler particles and interfaces within the composite structure. On the other hand, Vita Enamic samples (Figure 6G,H) demonstrated the most homogeneous microstructure, presenting a compact and interconnected matrix–filler network with fewer fracture-related defects. Gradia samples (Figure 6I,J) displayed less pronounced fracture-specific features and reduced surface fragmentation compared with the other materials.

### 3.7. Weibull Analysis

**Figure 7:**
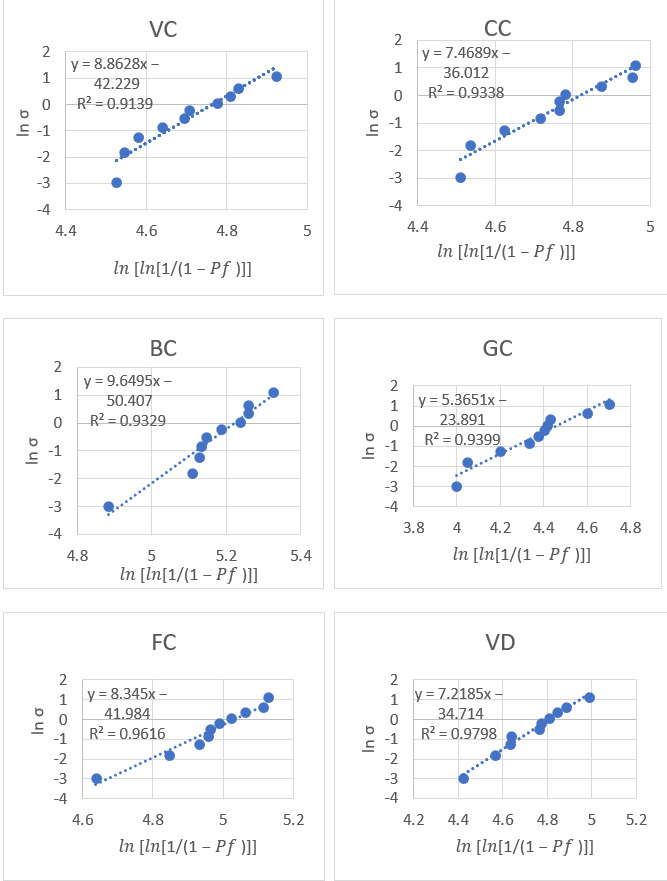

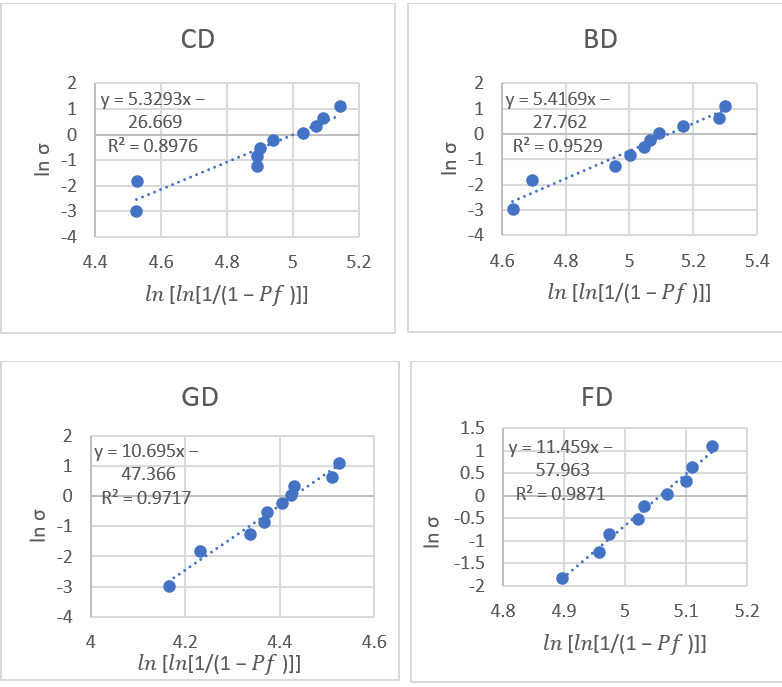
Weibull plots for each material.

A linearized Weibull plot was obtained for each material, for both the control and degraded groups, by graphically representing ln(σ) on the horizontal axis and *ln*[*ln*[1/(1 − *Pf*)]] on the vertical axis (Figure 7).

The two parameters, Weibull modulus obtained from the slope of the regression line and characteristic strength, calculated from the intercept, of each material in both the control and degraded groups are presented in Table 8. Also, the coefficient of determination r^2^ was used to assess how well the Weibull distribution fitted the experimental data [41]. Therefore, higher r^2^ values indicate a better fit, suggesting that the data more closely follow the Weibull distribution. In this research, the r^2^ values ranged between 0.89–0.98.

The Weibull modulus (m) values were in descending order: FD > GD > BC > VC > FC > CC > VD > BD > GC > CD. The characteristic strength (σ_0_) values were in descending order: BC > BD > FD > FC > CD > CC > VD > VC > GC > GD.

**Figure 8:**
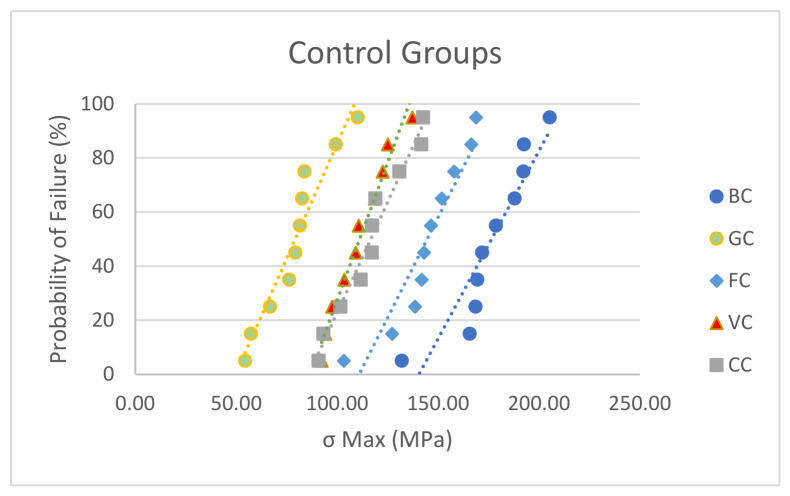
Probability of failure for the control groups.

**Figure 9:**
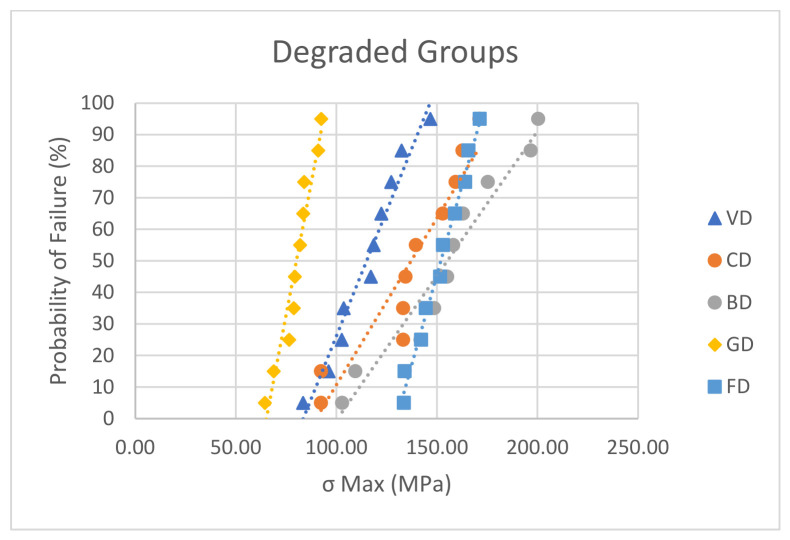
Probability of failure for the degraded groups.

Representative probability-of-failure plots for both the control and degraded groups are also presented in Figure 8 and Figure 9.

## 4. Discussion

The present research focused on two mechanical parameters that are well studied worldwide: flexural strength and elastic modulus. To measure these parameters, a three-point bending test was used, following ISO 6872, adapting the procedure to the size of the blocks [32].

Flexural strength refers to a material’s ability to resist bending when a load is applied, whereas the elastic modulus measures how rigid the material is when flexed [21]. In this study, the highest value of flexural strength was recorded for Brilliant Control (174.47), while the lowest was recorded for Gradia Control (79.2).

Regarding the elastic modulus, the literature has concluded that the force–displacement curves obtained from the three-point bending test are considered to be the most exact [41]. The highest value of the elastic modulus was registered by Vita Degraded (20.31), while the lowest was recorded for Gradia Control (5.8). Studies report that human enamel has an elastic modulus between 74 and 130 GPa, while dentin ranges from 17.7 to 29.8 GPa [34,35,36,37]. The elastic modulus values obtained in this study, ranging between 5.8 and 20.31 GPa, place these materials closer to natural dentin. Vita Enamic demonstrated the highest value of elastic modulus, so it’s the stiffest tested material. However, the overall lower values can be explained by the composition of the material tested. Because resin-based and hybrid ceramics include a polymer matrix, they present a tendency to be more flexible and less stiff in comparison with pure ceramics or enamel. From a clinical point of view, these materials behave more like dentin, a fact that is beneficial for stress support and avoidance of fracture. The need to measure this mechanical parameter is important, as clinically, a low elastic modulus may compromise the clinical outcome by causing restorative displacement and increasing wear [42,43]. Consequently, the ultimate significance of elastic modulus relates to the selection of the appropriate composite for a particular therapeutic context [44].

The internal structure of the matrix and filler inside a dental material determines its mechanical performance [42]. It is believed that a high amount of filler leads to improved mechanical parameters such as strength and stiffness, but also to enhanced aesthetics. Research indicates that there is a direct relationship between the amount of filler and the mechanical characteristics, with microfilled composites showing reduced values of mechanical parameters [17]. Among the studied materials, Vita Enamic, Cerasmart, Brilliant Crios, and Filtek present the highest filler content, which may explain their improved mechanical response. Vita Enamic is a hybrid material, representing the PICN technology (Polymer-Infiltrated Ceramic Network), in contrast to other CAD-CAM blocks created using composite filler-resin mixing technique (such as Cerasmart) [21]. Theories imply that the high temperature and pressure present during the curing process decrease the internal defects and voids present in the PICN microcomposition. In this way, fissure appearance and spread are avoided [43]. In one study, Vita Enamic presented high values of elastic modulus, but low values of flexural strength, result that was attributed to the fact that connecting the porous ceramic structure with the polymeric resin may be detrimental to bending strength [21]. This was supported by another research, where the elastic modulus was higher and the flexural strength lower than that of other CAD-CAM blocks [44]. Accordingly, these results were supported in this study, as Vita Enamic recorded the highest value of elastic modulus, while its flexural strength values were a bit lower than those of Cerasmart and Brilliant.

An important finding of this research was the high values registered for Filtek (a direct resin composite), which were close to those of the CAD-CAM materials. Filtek is a universal nano-hybrid resin composite. Silica oxide particles, modified zirconium and silicon oxides, and clustered aggregates constitute 82% of its inorganic component, while BIS-GMA, UDMA, BIS-EMA, PEGDMA and TEGDMA represent its organic matrix [45]. Consequently, this excellent result could be attributed to its filler composition consisting of nano-clusters, which would lead to increased mechanical strength [46]. The outcomes also suggest that zirconia-silica fillers, which are part of the Filtek structure, could have a higher mechanical strength than barium glass fillers [47].

The water-aging method was chosen because water sorption can negatively affect materials by dissolving the matrix, filler, and bonding surface, which in turn can impact their mechanical performance [48,49]. As water molecules enter the polymer network and diffuse between its intermolecular chains, swelling and changes in the material’s dimensions may occur [50]. Accordingly, the absorption of water can negatively influence the mechanical properties of resin composite materials by weakening the resin matrix and decreasing mechanical performance, translated into reduced strength and stiffness [51]. As a result, the longevity of the restoration may be compromised [51]. Both the kind and composition of the dental resin composite play an important role in determining water sorption. Composites with a greater filler loading usually absorb less water, as they contain less resin matrix accessible for water diffusion. Furthermore, the type of monomer is an important determinant. For example, monomers like Bis-GMA (bisphenol A-glycidyl methacrylate) tend to absorb more water because the hydroxyl groups in their structure promote interactions with water molecules [52].

Due to industrial polymerization, CAD/CAM resin composites develop a more densely crosslinked structure with fewer defects, such as pores and voids. Along with a higher filler content, these features can decrease water absorption and improve the material’s resistance to long-term chemical and mechanical degradation [42]. The overall good behaviour of the CAD-CAM resin composites in this study is sustained by the internal composition, the manufacturing process and also approved by the Weibull analysis.

In this study was observed in the case of some material, a slight increase, but not statistically significant of the flexural strength. This might be explained by the plastic region expanding before the fracture spreads and the tension being removed [48]. Consequently, the first null hypothesis is rejected, as there were statistically significant differences between materials. On the other hand, the second null hypothesis is accepted, as there were no statistically significant differences between the control and degraded groups (with the exception of Gradia). It can be concluded that the hydration–dehydration cycles did not statistically significant affect the mechanical properties. This finding was also reported in another study, where 30 days of water immersion had only a minor effect on the elastic modulus and flexural strength [32]. It has been demonstrated that cyclical fatigue has a stronger correlation with the flexural strength of composite resins over extended periods in water than simple storage does [35].

Fractography of dental materials has been neglected, despite the major role that crown fracture plays in the long-term clinical assessment of dental materials. It is known that both direct restorative materials (used as filling materials for cavity treatment—Gradia and Filtek investigated in the present study) or indirect materials (indirect restorative materials (used for crowns when clinically indicated—Cerasmart, Vita Enamic, Brilliant Crios), may suffer degradation over time, eventually leading to fracture, due to various mechanisms related to internal or external factors. Consequently, this study addressed this issue, by evaluating the effect of water storage on the mechanical properties of these materials, as well as its influence at the microstructural level through SEM analysis.

The materials investigated in this study are positioned at the boundary between the more ductile behavior of polymers, which are more sensitive to applied loading and may show deformation features such as crazing and the brittle behavior characteristic of ceramics [7]. However, literature states that filled resin composites have a brittle-like materials acting, characterized by approximately linear fracture patterns, as observed in in vitro studies [16]. From a fractographic perspective, the filler content is closely associated with the extent to which these materials display similarities to the fracture behavior of ceramics and glasses [16]. In this research, Vita Enamic (PICN material) demonstrated the greatest structural homogeneity and the fewest fracture-induced microstructural changes, probably due to its interconnected ceramic network. Also, SEM evaluation revealed fracture-specific features, including wake hackle lines, fracture origin, compression curls, river marking and crack propagation direction. Brilliant Crios, Filtek Z550 and Cerasmart showed the most notable fracture-related changes, while no significant microstructural differences were observed between the control and degraded samples. In conclusion, the third null hypothesis was rejected, as there were overall differences in the fractographic aspect.

Also, in terms of fractographic characteristics, the lower values of the mechanical parameters (elastic modulus and flexural strength) would imply a significantly lower amount of elastic energy accumulated before fracture, resulting in reduced fragmentation and less secondary crack formation [16]. The lowest values of both flexural strength and elastic modulus were recorded for Gradia Control, which may explain the less pronounced fracture-specific features observed in this material.

From the Weibull analysis it can be concluded that FD, which demonstrated the highest Weibull modulus (m) value (11.45) showed the highest reliability and the lowest scatter of flexural strength values, followed by GD, BC, VC, while CD (the lowest Weibull modulus values) displayed (the highest variability in strength and the lowest reliability, followed by GC and BD. Regarding the characteristic strength, BC demonstrated the highest characteristic strength value, followed by BD, FD and GD showed the lowest and therefore the lowest resistance to failure. The high values of r^2^ suggest a good fit between the Weibull distribution and the data. However, the relatively small sample size (n = 10) represents a limitation of the Weibull analysis. Therefore, the Weibull modulus results should be interpreted with caution.

The limitations of the present study and future perspective consist of: use of additional degradation protocols, such as thermocycling and UV aging, the assessment of parameters beyond mechanical properties, including color stability and surface roughness the evaluation of other mechanical tests such as cyclic loading, the use of higher SEM magnifications, and the investigation of additional aging methods and categories of materials, such as CAD/CAM materials fabricated through additive manufacturing technologies.

## 5. Conclusions

Within the limitations of this study, the following conclusions can be drawn:

1. The cycles of dehydration and hydration did not change the material’s mechanical characteristics (flexural strength and modulus of elasticity), as there were not recorded significant differences between control vs. degraded groups.
2. There were significant differences between the two categories of tested materials (direct resin composites vs. CAD-CAM resin composites), highlighting their distinct mechanical behavior in favor of the CAD-CAM category, which can be strongly explained by their internal structure.
3. The flexural strength values exceeded the minimum value of 80 MPa required to withstand masticatory forces, with the exception of one direct resin composite
4. The values of the elastic modulus demonstrated the similarity of these dental materials to natural human tissues and their beneficial effect on clinical outcomes.
5. A notable performance in terms of the flexural strength was recorded for the universal nano-hybrid direct resin composite, possibly explained by its high inorganic filler content organized in nano-clusters.
6. The Weibull analysis revealed greater reliability and lower data variability, translated through the highest Weibull modulus-m values, for the direct resin composites materials.
7. The Weibull analysis revealed generally high characteristic strength for the CAD-CAM materials, a notable performance in terms of characteristic strength being demonstrated also for the universal nano-hybrid direct resin composite. The micro-filled direct resin composite recorded the lowest characteristic strength.
8. The fractographic analysis revealed specific fracture features, with the material from the PICN category being noted as the most homogeneous and presenting the fewest fracture defects, probably due to its internal structure.

## Figures and Tables

**Table 1:** Direct resin composites used in this study.

Material	Manufacturer	Matrix Composition	Filler Type	Filler Content (%)
Gradia Direct Anterior A2 (G)	GC Corporation, Tokyo, Japan	UDMA, Bis-EMA, dimethacrylates, trimethacrylates	Barium glass, silica, prepolymerized resin fillers	69
Filtek Z550 A2 (F)	3M ESPE, USA	Bis-GMA, UDMA, TEGDMA, BIS-EMA, PEGDMA	Zirconia/silica nanoparticles	78.5

**Table 2:** CAD CAM materials used in this study.

Material	Type	Manufacturer	Filler	Monomer	Shade/Translucency
Vita Enamic (V)	Hybrid Ceramic	VITA Zahnfabrik, Bad Säckingen, Germany	Feldspar ceramic enriched with aluminum oxide 86%	UDMA, TEGDMA	A2/MT
Cerasmart (C)	CAD/CAM composite resin	GC Corportion Tokyo, Japan	Silica, barium glass 71%	UDMA, Bis-MEPP, DMA	A2/MT
Brilliant Crios (B)	CAD/CAM Composite resin	Coltene/Whaledent, Alstatten, Switzerland	Amorphous silica particles (<20 nm) and glassy ceramic, barium particles (<1.0 µm), 70.7%	BisGMA, UDMA, TEGDMA	A2

**Table 3:** *p*-value Tukey HSD pairwise comparison between two materials, control groups; VC—Vita Control, CC—Cerasmart Control, BC—Brilliant Control, GC—Gradia Control, FC—Filtek Control.

Materials		Mean Difference	SE	df	t	*p*-Value
VC-	CC	−5.529	8.068	45	−0.685	0.959
	BC	−65.374	8.068	45	−8.103	<0.001
	GC	31.896	8.068	45	3.954	0.002
	FC	−33.482	8.068	45	−4.150	0.001
CC-	BC	−59.845	8.068	45	−7.418	<0.001
	GC	37.425	8.068	45	4.639	<0.001
	FC	−27.953	8.068	45	−3.465	0.010
BC-	GC	97.270	8.068	45	12.057	<0.001
	FC	31.892	8.068	45	3.953	0.002
GC-	FC	−65.378	8.068	45	−8.104	<0.001

**Table 4:** *p*-value Tukey HSD pairwise comparison between two materials, degraded groups; VD—Vita Degraded, CD—Cerasmart Degraded, BD—Brilliant Degraded, GD—Gradia De-graded, FD—Filtek Degraded.

Materials		Mean Difference	SE	df	t	*p*-Value
VD-	CD	−22.104	9.733	45	−2.271	0.173
	BD	−40.108	9.733	45	−4.121	0.001
	GD	34.958	9.733	45	3.592	0.007
	FD	−36.871	9.733	45	−3.788	0.004
CD-	BD	−18.004	9.733	45	−1.850	0.359
	GD	57.062	9.733	45	5.862	<0.001
	FD	−14.767	9.733	45	−1.517	0.557
BD-	GD	75.066	9.733	45	7.712	<0.001
	FD	3.237	9.733	45	0.333	0.997
GD-	FD	−71.829	9.733	45	−7.380	<0.001

**Table 5:** *p*-value of E-Tukey HSD pairwise comparison between two materials, control groups; VC—Vita Control, CC—Cerasmart Control, BC—Brilliant Control, GC—Gradia Control FC—Filtek Control.

Materials		Mean Difference	SE	df	t	*p*-Value
VC-	CC	10.893	0.487	45	22.372	<0.001
	BC	10.070	0.487	45	20.680	<0.001
	GC	13.661	0.487	45	28.055	<0.001
	FC	8.503	0.487	45	17.462	<0.001
CC-	BC	−0.824	0.487	45	−1.692	0.449
	GC	2.768	0.487	45	5.684	<0.001
	FC	−2.391	0.487	45	−4.910	<0.001
BC-	GC	3.591	0.487	45	7.376	<0.001
	FC	−1.567	0.487	45	−3.218	0.019
GC-	FC	−5.158	0.487	45	−10.594	<0.001

**Table 6:** *p*-value of E-Tukey HSD pairwise comparison between two materials, degraded groups; VD—Vita Degraded, CD—Cerasmart Degraded, BD—Brilliant Degraded, GD—Gradia Degraded, FD—Filtek Degraded.

Materials		Mean Difference	SE	df	t	*p*-Value
VD-	CD	10.803	0.736	45	14.672	<0.001
	BD	11.055	0.736	45	15.015	<0.001
	GD	13.180	0.736	45	17.901	<0.001
	FD	9.608	0.736	45	13.049	<0.001
CD-	BD	0.252	0.736	45	0.342	0.997
	GD	2.377	0.736	45	3.229	0.019
	FD	−1.195	0.736	45	−1.623	0.491
BD-	GD	2.125	0.736	45	2.887	0.045
	FD	−1.447	0.736	45	−1.966	0.299
GD-	FD	−3.572	0.736	45	−4.852	<0.001

**Table 7:** Unpaired Student’s *t* Test for pairwise combinations of VC—Vita Control, CC—Cerasmart Control, BC—Brilliant Control, GC—Gradia Control FC- Filtek Control, VD—Vita Degraded, CD—Cerasmart Degraded, BD—Brilliant Degraded, GD—Gradia Degraded, FD—Filtek Degraded

Material	σ max—*p* Value	E—*p* Value
VC -VD	0.609	0.288
CC-CD	0.064	0.308
BC-BD	0.095	0.801
GC-GD	0.889	0.001
FC-FD	0.337	0.574

**Table 8:** Weibull modulus (m) and characteristic strength (σ_0_) of VC—Vita Control, CC—Cerasmart Control, BC—Brilliant Control, GC—Gradia Control, FC— Filtek Control, VD—Vita Degraded, CD—Cerasmart Degraded, BD—Brilliant Degraded, GD—Gradia Degraded, FD—Filtek Degraded.

Material	Weibull Modulus (m)	Characteristic Strength (σ_0_)	r^2^
VC	8.86	117.35	0.91
CC	7.46	124.84	0.93
BC	9.64	186.45	0.93
GC	5.36	86.23	0.93
FC	8.34	153.48	0.96
VD	7.21	123.24	0.97
CD	5.32	150.09	0.89
BD	5.41	169.22	0.95
GD	10.69	83.95	0.97
FD	11.45	157.9	0.98

## Data Availability

The original contributions presented in this study are included in the article. Further inquiries can be directed to the corresponding author.
